# The Key Ripper: a specialized fixed suture-cutting device to facilitate drain removal

**DOI:** 10.1007/s00595-024-02897-9

**Published:** 2024-07-20

**Authors:** Shogo Nagamatsu, Ayano Sasaki, Yumiwo Fujioka, Yoshiaki Shibata, Ken Nomimura, Yuki Aoki, Isao Koshima

**Affiliations:** 1 Department of Plastic and Reconstructive Surgery, Hiroshima University Hospital, Kasumi 1-2-3, Minami-Ku, Hiroshima, 734-8551 Japan; 2 International Centre for Lymphedema, Hiroshima University Hospital, Hiroshima, Japan

**Keywords:** Post-surgical drain removal, Key Ripper

## Abstract

The removal of post-surgical drains requires suture cutting. We developed a novel and specialized device that can safely remove the threads fixing the drains, making drain removal safer and easier than the current removal approach using scissors or a scalpel.

**Supplementary Information:**

The online version contains supplementary material available at 10.1007/s00595-024-02897-9.

## Introduction

Tubes such as drains and central venous catheters are essential in plastic surgery and other surgical and medical fields. Suturing these tubes to the skin with a silk thread is a widely accepted technique to secure placement [1–3]. When the drain is no longer needed, these threads must be cut to allow its removal. However, no specialized thread-cutting instruments are available for suture removal, which is usually performed using scissors or a scalpel with a risk of injury to the patient or damage to the drain. Thus, we designed a simple and novel device for the sole purpose of cutting the threads securing a drain. This device is safe because it only exposes the sutures to the covered blade thereby preventing injury to the patient and surgeon and damage to the drain. We describe the mechanism and discuss the results of this new device.

## Key Ripper development

**Fig. 1:**
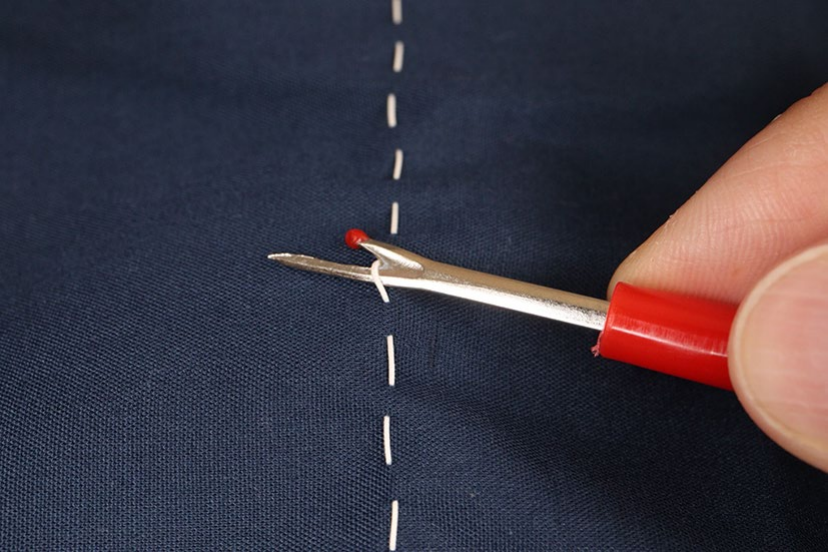
Sewing ripper used for cutting seams during sewing work

**Fig. 2:**
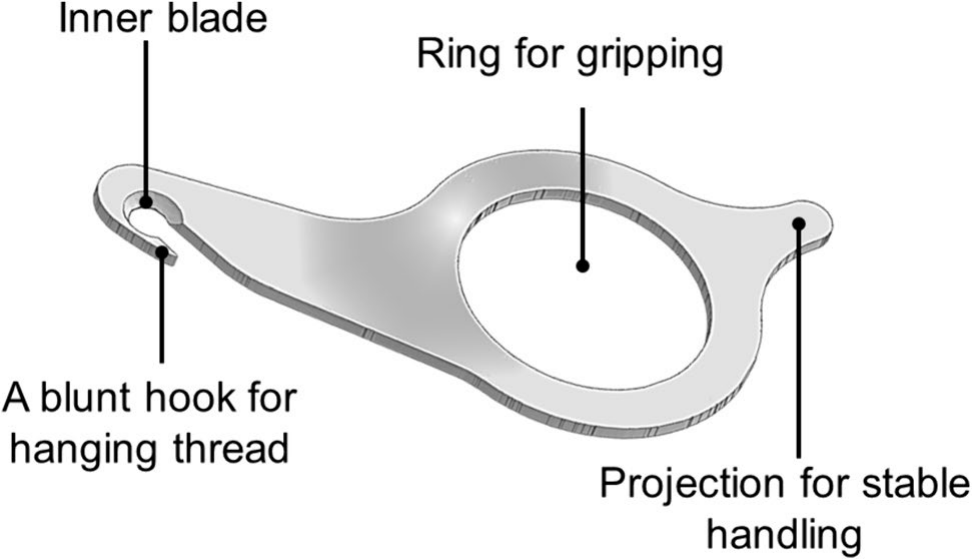
Structure and use of the “Key Ripper” for suture thread removal

**Fig. 3:**
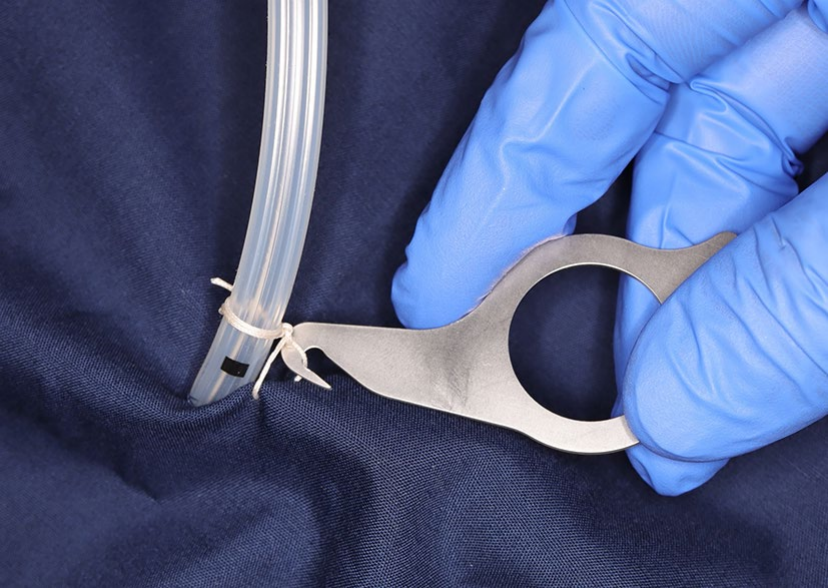
Animated diagram of the “Key Ripper” and its structures. Note that the blade edge faces inward, and the difficulty of cutting is based on its surroundings. The size of the device is 6 × 3 cm

The device, named the “Key Ripper,” resembles a key and is made of metal. It measures 6 × 3 cm and its design was inspired by a sewing ripper (Fig. 1). The Key Ripper has a ring-shaped handle and tip with a built-in blade. The tip of the instrument has a hook-shaped component for lifting the suture, as well as a completely inward-facing blade that prevents injury to the user even when accidentally touched or struck. The ring is grasped with the fingertips, and the hook at the tip is used to lift the suture, which is then cut with the blade inside (Figs. 2, 3; Video 1). Since its implementation in July, 2021, we have used this device to safely remove hundreds of drain tubes during daily ward procedures. The device has exhibited a 100% success rate for suture cutting, and scissors have never been required. Furthermore, there has been no accidental damage to the drain tubes or injuries to the patient or surgeon.

## Discussion

The Key Ripper is a simple device developed solely for suture thread cutting, making ward procedures such as drain removal safer and more efficient. The removal of drains and cannulas fixed to the body surface is essential in all medical fields. While various methods have been reported for fixing drain tubes, the most common of which is suturing them in place on the body surface using silk threads, to our knowledge, a device for cutting fixed threads has not been described before [1–6].

In most cases, sharp scissors or a scalpel are used or, if these are not available, a thick 18G injection needle may be used. Although scissors are extremely versatile, they are expensive and carry safety concerns related to loss or dullness over time. Furthermore, using a scalpel or injection needles carries a risk of injury to the patient and surgeon and of damage to the drain, if used incorrectly. Our small, simple, and key-like device makes drain removal safe and easy, eliminating the need for scissors or a scalpel.

This device has various advantages for daily drain removal. First, it is possible to remove the drain at an appropriate time without using scissors or a scalpel. Because surgical scissors can be expensive, they are often kept under strict control with limited accessibility, making them inconvenient. Preparing a sufficient number of Key Ripper devices can avoid this issue. Second, the mechanism of this device is simple and safe, allowing its use even by untrained personnel as it does not require maneuvering like scissors nor does it expose operators and patients to sharp blades, like a scalpel. As with sewing rippers, the thread can be lifted with a hook and cut safely with an inward-facing blade. We opted for a shape different from that of a sewing ripper to minimize the risk of accidentally piercing the patient’s skin with the tip after cutting the thread. By prioritizing operational safety, we designed the device to allow cutting by pulling it towards the user, thereby reducing the risk of injury. The proposed Key Ripper can be used easily even by older surgeons with presbyopia and those with limited dexterity, because it is designed specifically to cut only the thread.

Existing tools, such as scissors and scalpels, are highly versatile and widely used, but they are not specialized for cutting threads; therefore, there is room for improvement in terms of cost and safety. Such instruments will continue to be needed; however, the Key Ripper contributes to safe and efficient drain removal without wasting scissors or scalpels. To date, no other device has been designed specifically to safely cut only the threads that secure drains. The Key Ripper consists of a handle made from a single metal plate, which is easy to hold and operate, as well as a tip with a securely built-in blade. When using this device, only the thread is lifted and guided to the blade, thereby eliminating the risk of cutting anything other than the thread. The device can also be used for other routine suture removal procedures.

The Key Ripper device is limited by its implementation. Proper use requires an appropriate amount of slack in the sutured thread to allow space for placement of the lifting hook. Typically, sutures fixing drain tubes are not tied tightly, to prevent them from digging into the skin, and some slack usually exists. These sutures can be removed using scissors or similar devices without compromising the drain’s secure placement. Thus, there is no need to change the traditional drain-fixing method when using the Key Ripper. Furthermore, when using older instruments, thread cutting may take longer because of dulled blades, causing the patient discomfort. We are continuously improving the sharpness and efficacy of our tool to ensure a painless experience for the patient.

Currently, the cost to manufacture the Key Ripper is about 2000 yen (approximately $12.84) per device. We believe that further cost reductions are possible by optimizing the manufacturing process and reevaluating the shape and materials used. For comparison, the cost of one pair of ophthalmic scissors is about 5000 yen (approximately $32.11). If the Key Ripper can be improved in quality and mass-produced at a low cost, it will improve medical care, lower costs, and improve safety. We hope that this safe and simple device will be implemented in clinical settings worldwide.

## Supplementary Information

Below is the link to the electronic supplementary material.

**Video 1**. Video of the “Key Ripper” cutting the threads securing various tubes (MPG 6096 KB)

## References

[1] Ringel Y, Haberfeld O, Kremer R, Kroll E, Steinberg R, Lehavi A. Intercostal chest drain fixation strength: comparison of techniques and sutures. BMJ Mil HealTH. 2021;167:248–50. 33093024 10.1136/bmjmilitary-2020-001555

[2] Heskin L, Cahill V, Filobbos G, Regan P, O’Sullivan ST, Bryan K. A new adaptation for a secure surgical drain placement and a comparison with four common drain fixation methods. Ann R Coll Surg Engl. 2019;101:60–8. 30328703 10.1308/rcsann.2018.0177 PMC6303816

[3] Inzirillo F, Giorgetta C, Ravalli E, Della PC. “Roman Sandal” modified method for securing the chest drain to the skin. Gen Thorac Cardiovasc Surg. 2013;61:171–3. 23188514 10.1007/s11748-012-0184-2

[4] Akuetteh PDP, Akuetteh YB, Shen X, Zhang Q, Zeng Q. A novel silicone fixation dressing: a possibly ideal method for drainage tube fixation. Surg Innov. 2020;27:644–6. 32677864 10.1177/1553350620942369

[5] Lauder CI, Strickland A, Maddern GJ. A novel technique for biliary T-tube fixation. Ann R Coll Surg Engl. 2010;92:169. 20364446 10.1308/003588410X12628812459058c PMC3025235

[6] Payne-James JJ, Savage PT. Surgeon’s workshop. A method of tube drain fixation. J R Coll Surg Edinb. 1987;32:39. 3560016

